# Fusarium head blight monitoring in wheat ears using machine learning and multimodal data from asymptomatic to symptomatic periods

**DOI:** 10.3389/fpls.2022.1102341

**Published:** 2023-01-16

**Authors:** Ghulam Mustafa, Hengbiao Zheng, Wei Li, Yuming Yin, Yongqing Wang, Meng Zhou, Peng Liu, Muhammad Bilal, Haiyan Jia, Guoqiang Li, Tao Cheng, Yongchao Tian, Weixing Cao, Yan Zhu, Xia Yao

**Affiliations:** ^1^ National Engineering and Technology Center for Information Agriculture, Key Laboratory for Crop System Analysis and Decision Making, Ministry of Agriculture, Jiangsu Collaborative Innovation Center for Modern Crop Production, Nanjing Agricultural University, Nanjing, China; ^2^ National Engineering and Technology Center for Information Agriculture, Jiangsu Key Laboratory for Information Agriculture, Ministry of Agriculture, Jiangsu Collaborative Innovation Center for Modern Crop Production, Nanjing Agricultural University, Nanjing, China; ^3^ Crop Genomics and Bioinformatics Center and National Key Laboratory of Crop Genetics and Germplasm Enhancement, Nanjing Agricultural University, Nanjing, Jiangsu, China

**Keywords:** fusarium head blight, asymptomatic detection, sequential floating forward selection, machine learning classifier, disease estimation, multimodal data

## Abstract

The growth of the fusarium head blight (FHB) pathogen at the grain formation stage is a deadly threat to wheat production through disruption of the photosynthetic processes of wheat spikes. Real-time nondestructive and frequent proxy detection approaches are necessary to control pathogen propagation and targeted fungicide application. Therefore, this study examined the ch\lorophyll-related phenotypes or features from spectral and chlorophyll fluorescence for FHB monitoring. A methodology is developed using features extracted from hyperspectral reflectance (HR), chlorophyll fluorescence imaging (CFI), and high-throughput phenotyping (HTP) for asymptomatic to symptomatic disease detection from two consecutive years of experiments. The disease-sensitive features were selected using the Boruta feature-selection algorithm, and subjected to machine learning-sequential floating forward selection (ML-SFFS) for optimum feature combination. The results demonstrated that the biochemical parameters, HR, CFI, and HTP showed consistent alterations during the spike–pathogen interaction. Among the selected disease sensitive features, reciprocal reflectance (RR=1/700) demonstrated the highest coefficient of determination (*R* ^2^) of 0.81, with root mean square error (RMSE) of 11.1. The multivariate k-nearest neighbor model outperformed the competing multivariate and univariate models with an overall accuracy of *R* ^2^ = 0.92 and RMSE = 10.21. A combination of two to three kinds of features was found optimum for asymptomatic disease detection using ML-SFFS with an average classification accuracy of 87.04% that gradually improved to 95% for a disease severity level of 20%. The study demonstrated the fusion of chlorophyll-related phenotypes with the ML-SFFS might be a good choice for crop disease detection.

## 1 Introduction

Among the biotic stress challenges to wheat cereals, fusarium head blight (FHB) has been causing extensive and severe damage to wheat crops since the early 20th century (McBeath and McBeath, 2010). FHB is equally detrimental to humans and livestock because it produces fungal mycotoxins and causes discoloration, weight reduction, and production, quality and yield losses (Bauriegel et al., 2011). Therefore, early and real-time detection and monitoring is a potential option for controlling FHB (Zhang et al., 2020). For this purpose, reflectance and chlorophyll fluorescence-based imaging (Multispectral and hyperspectral images, Chlorophyll fluorescence images, etc.) and non-imaging (Multispectral and hyperspectral reflectance or spectroscopy) sensors are being employed successfully for plants’ disease monitoring (Bauriegel et al., 2011; Bauriegel and Herppich, 2014; Mahlein et al., 2019).

The FHB pathogen deteriorates internal pigmentation and physiological structure during the plant–pathogen interaction, which can be observed by reflectance spectroscopy (Kuenzer and Knauer, 2013). In agricultural remote sensing, reflectance spectroscopy is considered a competitive high-throughput phenotyping tool (Araus Ortega et al., 2018). Few studies have examined the spike–pathogen interaction using reflectance spectroscopy. For example, Ma et al. (2020) studied the reflectance of FHB, applied wavelet transforms and combined with Fisher linear analysis to measure the spectra from an angle to the side of wheat ears, and developed an identification model with an overall 88% accuracy. Likewise, (Huang et al., 2019a) used Fisher analysis with support vector machine (SVM) classification to develop a discriminant model. In addition, hyperspectral analyses have been successfully implemented in several crops for disease identification (Ren et al., 2021). Some studies have also explored hyperspectral imaging spectroscopy for FHB identification (Jin et al., 2018; Mahlein et al., 2019). These studies have indicated reflectance spectroscopy as an excellent candidate for spike studies. The numerous reflectance analysis approaches, for example, both narrow and broad bands (Thenkabail et al., 2004; Oumar et al., 2013), spectral derivatives (Gong et al., 2002), and transformed spectral reflectance (Zhao et al., 2021) are used. However, the application of vegetation indices (VI) is a simple and effective tool for detecting spectral variations (Ren et al., 2021). So far, the consistent sensitivity of VI in different years for FHB has yet to be investigated using spectral data.

Anatomically, the photosynthetic structure is primarily and severely affected by the hemi-biotrophic behavior of FHB (Kheiri et al., 2019). Thus, the net photosynthetic rate (Pn) is highly sensitive and could also be the best marker of pathogen invasion. The chlorophyll fluorescence spectroscopy is also an excellent approach for detecting plants’ early or real-time abiotic and biotic stress responses (Harbinson, 2013). Multiple fluorescence imaging techniques are used to investigate plant responses *via* different excitation modes. For example, anthocyanin levels in strawberry leaves have been estimated using UV light-induced fluorescence imaging of both the chlorophyll and blue-green fluorescence signals under *Nicotiana benthamiana* damage (Pineda et al., 2008). Kinetic fluorescence has been employed to examine *Arabidopsis* for drought tolerance and freeze-thaw (Ehlert and Hincha, 2008), virus infection in plants (Lei et al., 2017), and wheat responses to salt stress (Mehta et al., 2010). Chlorophyll fluorescence imaging (CFI) has also been applied for FHB detection and classification in combination with other remote sensors for wheat crops (Bauriegel et al., 2011; Mahlein et al., 2019), and to analyze pathogen severity on wheat spikes and leaves (Tan et al., 2021). However, the consistent sensitivity of CFI under different light excitation modes in different years for FHB remains to be investigated using machine learning (ML) approaches.

A comprehensive and temporal investigation of plants using remote sensors results in a huge dataset to compute output. Thus, for target output and data redundancy, ML helps through feature selection to select a subset of relevant features from the initially available dataset (Long et al., 2019). The mathematical models are classifiers from ML: a system that learns from given multiclass data and labels test data points (Wei et al., 2022). Numerous studies have used ML classifiers for disease detection, and they have become a valuable and widely applied mathematical tool in remote sensing studies (Zarco-Tejada et al., 2018).

Most of the previously conducted studies used all features (spectral and fluorescence) or biochemical/biophysical attributes to disease classification or regression models, regardless of the number of input variables. Many researchers have found that the amount of input variables or spectral features affect ML algorithms’ performance (Fallahpour et al., 2017; Bhardwaj and Patra, 2018). ML classifiers were used with feature selection techniques to improve fluorescence spectroscopic nucleotide identification (Huang et al., 2019b). Their machine learning and sequential floating forward selection (ML-SFFS) approach has not been applied to reflectance spectroscopy in combination with chlorophyll fluorescence of plants for disease diagnosis. The relative importance of each input indicator may vary by disease severity (DS) stage (Zarco-Tejada et al., 2018; Poblete et al., 2020). Thus, it is ambiguous how the partial fusion or combination of numerous spectral and fluorescence features improves FHB disease identification at different DS stages. FHB photosynthetic fingerprints on wheat spikes are rarely described in terms of net photosynthesis and chlorophyll concentration (Mustafa et al., 2022). Hence, the study conducted examination of wheat spikes pursuing principal objectives: (1) to determine highly disease-sensitive features (DSF) employing chlorophyll fluorescence imaging (CFI) and chlorophyll-related hyperspectral indices using a variable importance measure, and (2) to assess the ML-SFFS approaches focusing the multimodal data fusion for classification and estimation of disease at different levels of disease severity.

## 2 Materials and methods

### 2.1 Study site and plant material

**Figure 1:**
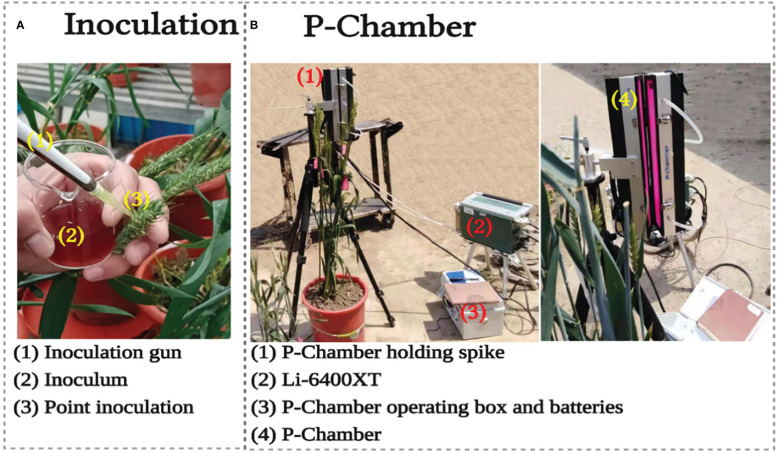
Spike FHB inoculation **(A)** and spike photosynthesis measurement with P-Chamber **(B)**.

The glasshouse-based winter wheat experiments were conducted in Jiangsu Province, China, for two consecutive seasons (2019–20 and 2020–21). The hyperspectral reflectance (HR) measurements were performed at the Pailou experiment base of Nanjing Agricultural University (Qinhuai District, Nanjing – 32°1’ N, 118°15’ E), and the fluorescence experiments were conducted at the Intelligent Glasshouse of Nanjing Agricultural University (Xuanwu District, Nanjing – 32°1’ N, 118°12’ E). HR plant material using two wheat varieties (Aikang-58 as susceptible and Sumai-3 as resistant to FHB) was grown successfully in 24 pots (size: 30 cm × 25 cm) in both growing seasons (2019–20 and 2020–21). The detail of the experiment material is given in **Table S1**. In each pot, 10 seeds were uniformly grown and managed following the method of Abid et al. (2017), where 12 pots were devoted to each variety and further halved to six for healthy and six for diseased plants. A similar protocol was followed for CFI that was identical to the HR plant material. Whereas, for high throughput phenotyping (HTP), seven wheat cultivars were grown: (1) Bainong-418, (2) Zhongyou-9507, (3) Jimai-31, (4) Wenmai-6, (5) Chianmai-42, (6) Huangpei-R4 as susceptible, and (7) Sumai-3 as resistant to FHB. In total, 56 pots were grown, seven of which were allocated to each cultivar, and five out of seven were inoculated (**Figure 1A**).

#### 2.1.1 Inoculation

The pots for the three types of sensors were inoculated with a freshly obtained inoculum of *Fusarium graminearum* from the State Key Laboratory of Crop Genetics and Germplasm Enhancement of Nanjing Agricultural University. The inoculum suspension of 2.5×10^5^ spores ml^−1^ was point inoculated for each spike in the middle spikelet (**Figure 1A**). The environment of all the plants was made favorable for successful fungal growth with high humidity, temperature 25–30°C and 16/8 hours of light/dark photo-period (Zhang et al., 2020). The inoculation was made at the growth stage (GS) 61–65 or flowering stage, where all spikes of uniform height and phenotype were inoculated in each pot.

#### 2.1.2 Disease severity

DS is the ratio of the symptomatic area to the asymptomatic area of the sample (Stack and McMullen, 1998). Due to the nonuniform development of disease infection, we designated nine different categories of DS: (1) asymptomatic (healthy), (2) DS1 (1–3%), (3) DS2 (4–5%), (4) DS3 (6–10%), (5) DS4 (11–20%), (6) DS5 (21–40%), (7) DS6 (41–60%), (8) DS7 (61–80%), and (9) DS8 (81–100%). The infection ratio or percentage of 4 infected ears from each pot was calculated based on the number of pixels using the Image J software package following Easlon and Bloom (2014).

### 2.2 Data measurement

#### 2.2.1 Spike photosynthesis measurement and chlorophyll content analysis

The Pn of the spikes was measured using a newly developed P-Chamber (**Figure 1B**) integrated with a portable photosynthesis system (LI-6400XT, Li-Cor Inc., Lincoln, NE, USA). The P-Chamber’s dimension is 30 cm × 5cm × 5cm (L × W × H), equipped with double-sided red and blue LED light source, and operate over a wide range of temperature (0–50°C) and humidity (0–95%) without condensation. A CO_2_ flow rate of 800 L min^−1^ was maintained due to the large size of the P-chamber. Further details of the experimental setup can be found *via* info@phenotrait.com and in Chang et al. (2020).

For spike chlorophyll contents (SCC), each spike was divided into three segments (upper, middle, and lower) and the parts (rachis, rachilla, glumes, lemma, palea, and awns) were mixed using a mortar and pestle. Then, 0.1 g of material was weighed out and stored in a vial containing 25 mL of ethanol (95%) for 48 h, till it turned white. The filtered samples were then placed in a 4.5 mL cuvette and their absorbance was measured at 470, 649, and 665 nm using a UV-visible spectrophotometer (Thermo Scientific Evolution 220, Thermo Scientific, Waltham, MA, USA). Afterwards, calculated the chlorophyll content using a Lichtenthaler (1987) standardized technique.

#### 2.2.2 Hyperspectral reflectance measurements

**Figure 2:**
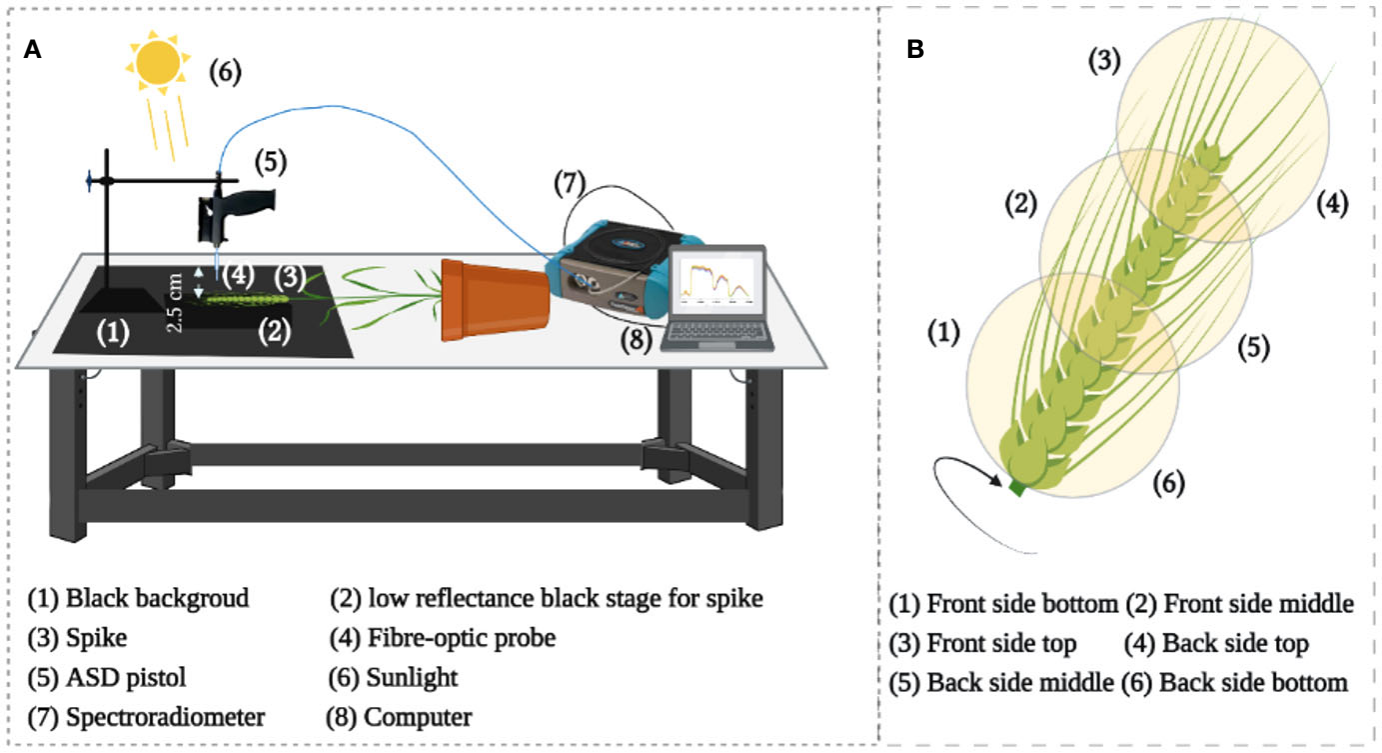
**(A)** Illustration of the setup for hyperspectral measurement, **(B)** Reflectance acquisition points from the whole individual spike.

For HR, a high-resolution spectroradiometer (ASD FieldSpec 4 Hi-Res, Malvern Panalytical, Westborough, MA, USA) was used to measure the spike HR with a sample interval of 1.4 nm in the 350-1000 nm region and of 1.11 nm in the range of 1001-2500 nm. The light reflected from the target was captured using a 1.5 m fiber optic contact wire and the ASD FieldSpec 4 Hi-Res array detector. Using a fiber optic probe, we observed the sample stage from a vertical position at sample-to-probe distance of approximately 2.5 cm using sunlight (**Figure 2A**) between 11:00 h and 14:00 h (Beijing time). In particular, all measurements were made non-destructively using same spikes on sunny days. In total, 40 spikes were measured for each year of the two-years experiments. In the end, five spectra were captured spatially from each position – the top, middle, and bottom of each spike from the front and back sides (**Figure 2B**). Eventually, 30 spectra were collected from each spike for subsequent analysis. This study used chlorophyll-related spectral indices (Zarco-Tejada et al., 2018; Tian et al., 2021) (**Table 1A** and **Table S2**).

**Table 1:** Variables included for fusarium head blight detection and estimation in the current study.

(A) Chlorophyll-related spectral indices				
	Chlorophyll indices	Abbreviations	Formulas	References
1	Reciprocal Reflectance	RR	1/R_700_	Gitelson et al. (1999)
2	Pigment Specific Simple Ratio	PSSRb	R_800_/R_650_	Blackburn (1998b)
3	Ratio Analysis of Reflectance Spectra	RARSb	R_675_/(R_675_×R_700_)	Chappelle et al. (1992)
4	Normalized Difference Vegetation Index	NDVI	(R800-R670)/(R800+R670)	Rouse et al. (1974)
5	Pigment Specific Normalized Difference	PSNDa	(R_800_-R_675_)/(R_800_+R_675_)	Blackburn (1998a)
6	Carter indices	CAR	R_695_/R_760_	Carter (1994)
A detailed description of all used spectral indices is given in **Table S2**.				
(B) Chlorophyll fluorescence variables				
	**Chlorophyll fluorescence variables – description**		**Abbreviations**	
1	Minimum fluorescence in dark-adapted state		F_0_	
2	Maximum fluorescence in dark-adapted state		F_m_	
3	Steady-state maximum fluorescence in light		F_m__Lss	
4	Fluorescence decline ratio in steady-state		Rfd_Lss	
5	Peak fluorescence during the initial phase of the Kautsky effect		fp	
6	Steady-state non-photochemical quenching		NPQ_Lss	
7	Steady-state PSII quantum yield		QY_Lss	
8	Maximum PSII quantum yield		QY = fv/fm	
(C) High-throughput phenotyping variables				
	**High-throughput phenotyping variables – description**		**Abbreviations**	
1	Red band image		R	
2	Green band image		G	
3	Blue band image		B	
4	Color image		Hue	
5	Color image		Saturation	
6	Color image		Value	
7	Photosynthetic efficiency of photosystem II image		Fv/Fm	
8	Chlorophyll image		Chl	
9	Chlorophyll index image		CHL.Index	
10	Anthocyanin reflectance index image		Ari.Index	
11	Normalized difference vegetation index image		NDVI	

#### 2.2.3 Chlorophyll fluorescence imaging

**Figure 3:**
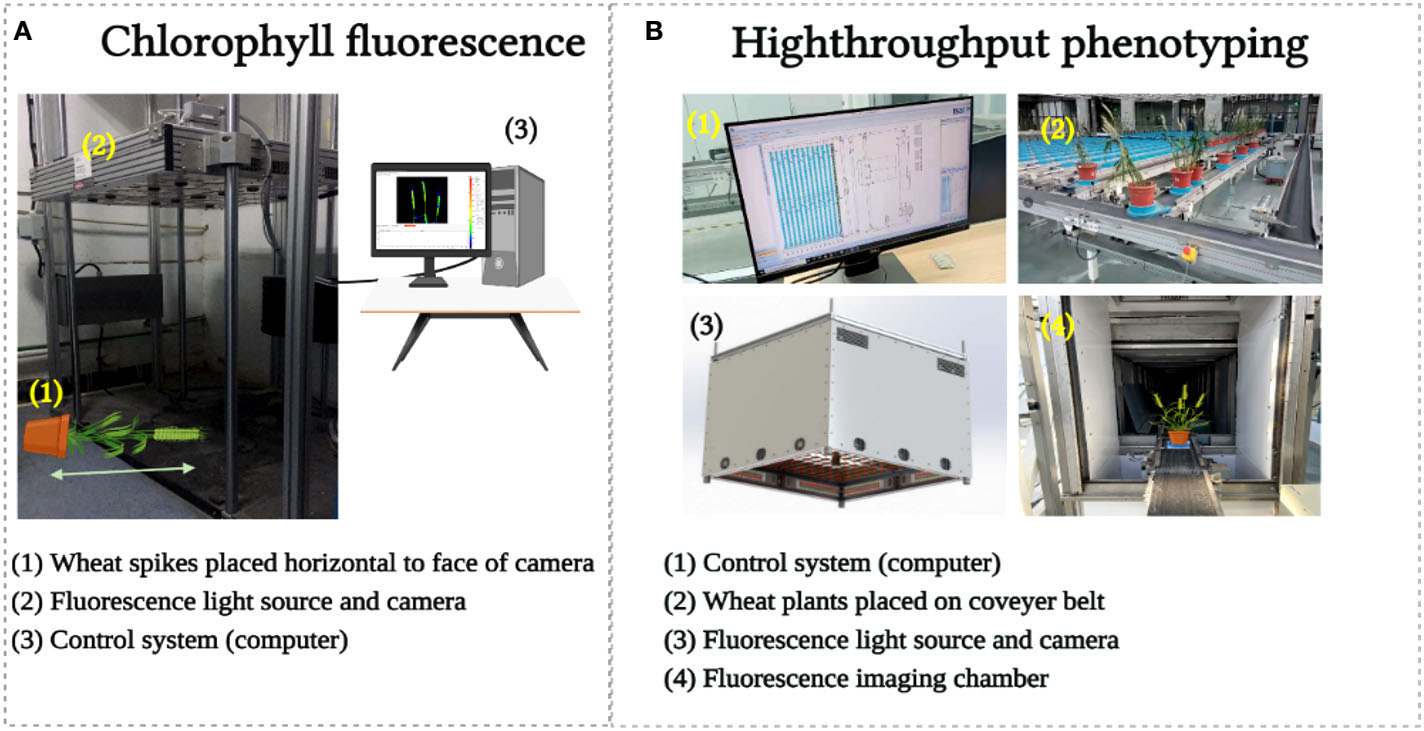
Chlorophyll fluorescence image acquisition **(A)** and high throughput phenotyping image acquisition **(B)**.

For CFI, an open FluorCam FC 800-O kinetic imaging fluorometer (PSI, Brno, Czech Republic) (**Figure 3A**) in which the light flashes for measurement of modulated CF excitation are produced by a pair of saturating light pulses (1 s, ~2000 μmol m^−2^ s^−1^) with red (λ_max_ ~618 nm) and blue LED panels (λ_max_ ~455 nm) producing actinic light. A charge-coupled device camera (CCD) with 12-bit resolution capturing 96 pixels per inch was employed to capture the CF kinetics at a frequency of 10 images per second (Granum et al. (2015). The spike pot was laid horizontally for precise exposure of the spike face toward the fluorescence camera (**Figure 3A**) and the same marked side was imaged daily. The spike’s region of interest (ROI) was cropped in FluorCam7 (PSI) software to obtain spike measurements as one biological sample. In total, 25 and 85 spikes were measured over the two time periods of the experiments in 2019–20 and 2020–21, respectively. **Table 1B** provides the details of selected variables as explained by the system developers.

#### 2.2.4 High-throughput phenotyping

For HTP, a nondestructive fluorescence and multispectral phenotyping platform were employed (CropReporter, PhenoVation B.V., Wageningen, the Netherlands) to monitor various real-time physiological traits. This platform acquired data *via* specific absorption, fluorescence, and reflection patterns in the visible (VIS) and near-infrared (NIR) wavelength ranges. The entire setup was automated (**Figure 3B**), while the core fluorescence and spectral image acquisition camera comprised a CCD, 16-bit camera, and fluorescence lights mounted on robotic cartesian coordinates. In total, 20 plants of each variety were imaged, and afterward, the measurements of the spike areas were acquired using ROI for subsequent data analysis. The system’s developers have explained the details of the extracted variables (**Table 1C**), and data were analyzed using the default software Data_Analysis_V562; a detailed description can be found in the study of Meng et al. (2020).

### 2.3 Algorithmic methodology for disease detection

The study selected disease sensitive features using Boruta, then after variance inflation factor (VIF) analysis, the partial fusion of selected disease sensitive features (SDSF) was made through ML-SFFS.

#### 2.3.1 Feature selection

The study selected DSF using the Boruta method. This wrapper approach uses random forest (RF) ensemble learning in which the relevant features are chosen by comparing the importance of the original attributes to randomly obtained important features *via* permuted copies. The main idea is: Random variables are made from the system copies. Then, the original system variables are compared to previously produced randomized variables to determine their value. Variables with larger importance are considered important (Kursa and Rudnicki, 2010). Regarding the color scheme of boxplots, green represents important features, yellow labels represent tentative features (score is close to the best shadow feature), red confirms feature rejection, and blue denotes shadow features. For each boxplot, the topmost edge, black line, and bottommost edge of the box denote the upper (Q3), median (Q2), and lower (Q1) quartiles, respectively. While, whiskers denote the maximum (Q3 + 1.5*IQR) and minimum (Q1–1.5*IQR) values defined through interquartile ranges (IQR = Q3-Q1), respectively. The circles outside boxplot denote the outliers. This study carried out this analysis using the Boruta package in the R-environment.

#### 2.3.2 Classification of FHB

A preliminary VIF analysis was made on the DSF – a subset selected following Boruta analysis for hyperspectral reflectance, chlorophyll fluorescence imaging, and high-throughput phenotyping features. Among these, the features with VIF of less than 10 were retained for subsequent analysis (Tian et al., 2021) and stated finally as “selected DSF” (SDSF). Thereafter, assuming the optimality of the SDSF and reducing the computational complexity, a sequential floating forward selection (SFFS) was integrated with machine learning classification (MLC) algorithms to develop optimal feature combination (Huang et al., 2019b). SFFS is a bottom-up search procedure developed by Pudil et al. (1994), which initiates the exploration of a null or random subset and selects the highly significant feature. The three MLCs: k-nearest neighbor (K-NN) (Weinberger et al., 2006), RF (Belgiu and Drăguţ, 2016), and SVM (Chang and Lin, 2001). We performed these analyses using the mlxtend package on a Jupyter notebook.

#### 2.3.3 Estimation of disease severity

The SDSF and DS were linked using univariate regression to derive empirical linear and multivariate regression (RF, SVM, and K-NN). The first-year (2019–2020) and second-year (2020–2021) datasets were used to develop and validate the regression models. Herein, the root mean square error (RMSE) – Eq. 1 (Where, *P_i_* and *O_i_* symbolize the predicted and measured values, respectively, and n denote the number of samples.) – and the coefficient of determination (*R^2^*) – Eq. 2 (Where, *ŷ_i_* represents points in the regression line or prediction, *ȳ* represents the mean of all values, *y_i_* symbolize the actual values and n denotes the number of samples or points) – were used to assess their predictive performance.


(1) $$RMSE=\sqrt{\frac{1}{n}\sum_{i=1}^{n}{\left(P_{i}-O_{i}\right)}^{2}}$$

(2) $$R^{2}=1-\frac{\sum_{i=1}^{n}{\left({\hat{y}}_{i}-y_{i}\right)}^{2}}{\sum_{i=1}^{n}{\left(y_{i}-{\bar{y}}_{i}\right)}^{2}}$$

Where, the classification performance was measured through the attributes of the confusion matrix and results are presented as overall accuracy (Eq. 3) (Gorunescu, 2011). Be noted, we practiced, supervised binary classification.


(3) $$Overall\;accuracy=\frac{TP+TN}{TP+TN+FP+FN}$$

Where, TP (in actual infected and model also predicted so), TN (in actual healthy model predicted same), FP (in actual healthy but model predicted infected) and FN (in actual infected but model predicted healthy).

## 3 Results

### 3.1 Biochemical, fluorescence, and spectroscopic changes under FHB invasion

**Figure 4:**
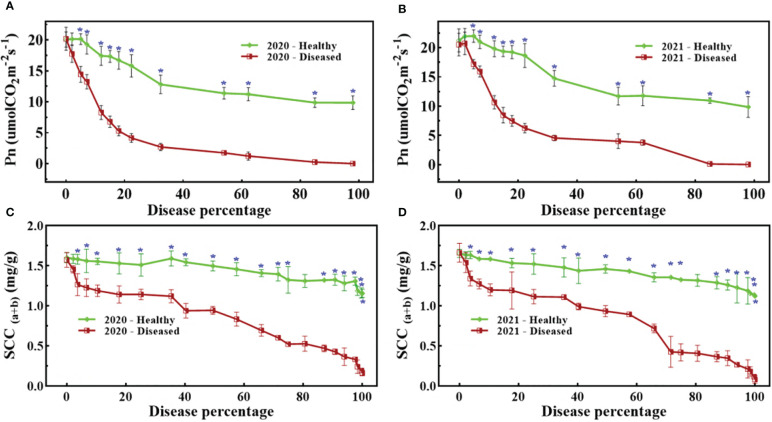
Dynamic changes of Pn **(A, B)** and SCC **(C, D)** against disease percentage in 2020 **(A, C)** and 2021 **(B, D)**. The blue asterisks mention the stage when there was statistical significance (t-test) between healthy and diseased spikes.

**Figure 4** shows that both SCC and Pn were severely affected by pathogen infection, but unexpectedly, the study also observed that healthy spikes also showed a slightly decreasing trend. All trends exhibited a noticeable fall in biochemical parameters due to pathogen infestation, but statistically significant differences were not common (**Figure 4A**). A statistically significant difference for Pn appeared at 5% disease percentage (DP) for the period 2019–20, while for the following period (2020–21) it appeared at 6% DP (**Figure 4B**). A similar trend can also be seen in **Figure 4C**, where a statistically significant difference appeared at 3% DP, while in **Figure 4D**, it appeared at 4% DP. In nutshell, the pathogen severely affected the biochemical parameters, but SCC were more sensitive than Pn.

**Figure 5:**
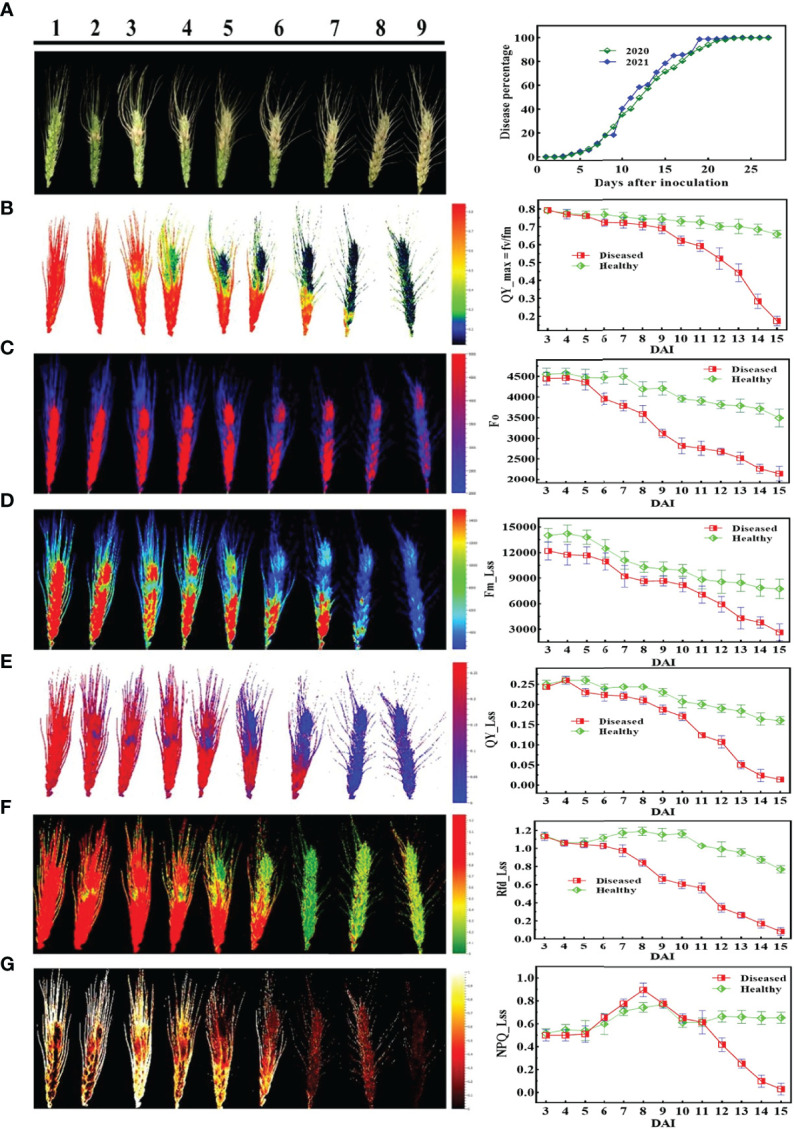
Illustration of the chlorophyll fluorescence features involved in the study to detect fusarium head blight (FHB): **(A)** RGB image of nine different disease severity (DS) and **(B-G)** are chlorophyll fluorescence parameters.

**Figure 6:**
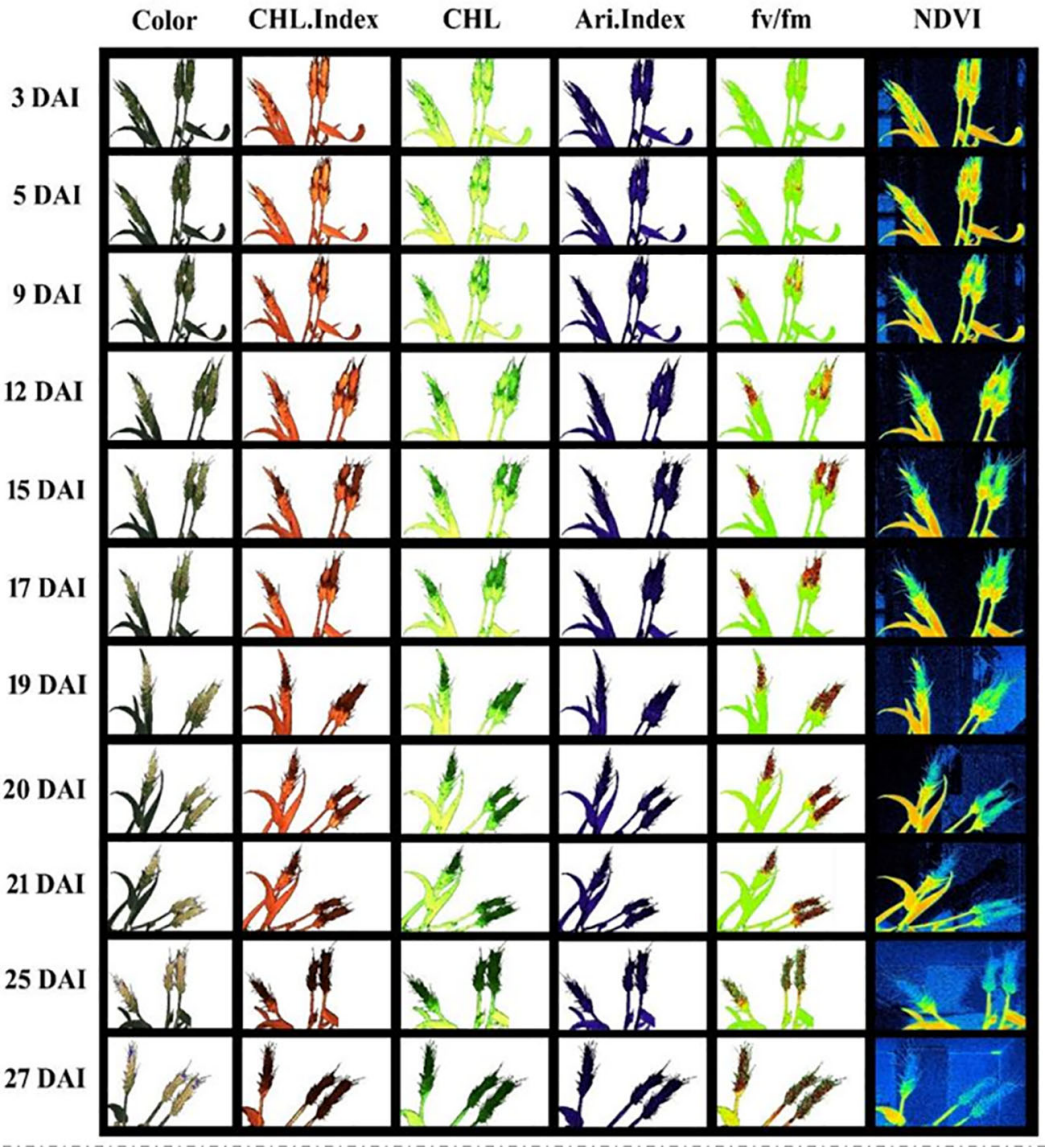
Spectral and fluorescence (left to right) response of wheat spikes under fusarium head blight (FHB) infection through high throughput phenotyping setup regarding days after inoculation (DAI) – top to bottom.

**Figures 5**, **6** demonstrate the photosynthetic fingerprints of FHB disease invasion on wheat spikes for CFI and HTP, respectively. The DP in respect of days after inoculation (DAI) for two years is shown in the **Figure 5A**. In **Figure 5**, QY showed the clear difference between healthy and diseased samples from 3DAI. Likewise, the Fm_Lss demonstrated the significant difference between healthy and disease spikes, but the F_o_ showed a balanced response until 5 DAI. However, NPQ responded in absolutely different manner in comparison to all other parameters, it showed first resistance and remained consistent until 5DAI but from 6 to 10 DAI a clear rise in diseased plants was depicted. The HTP shows the clear change (pictorial form-data not shown) in the ears for fv/fm, and CHL.Index (**Figure 6**).

**Figure 7:**
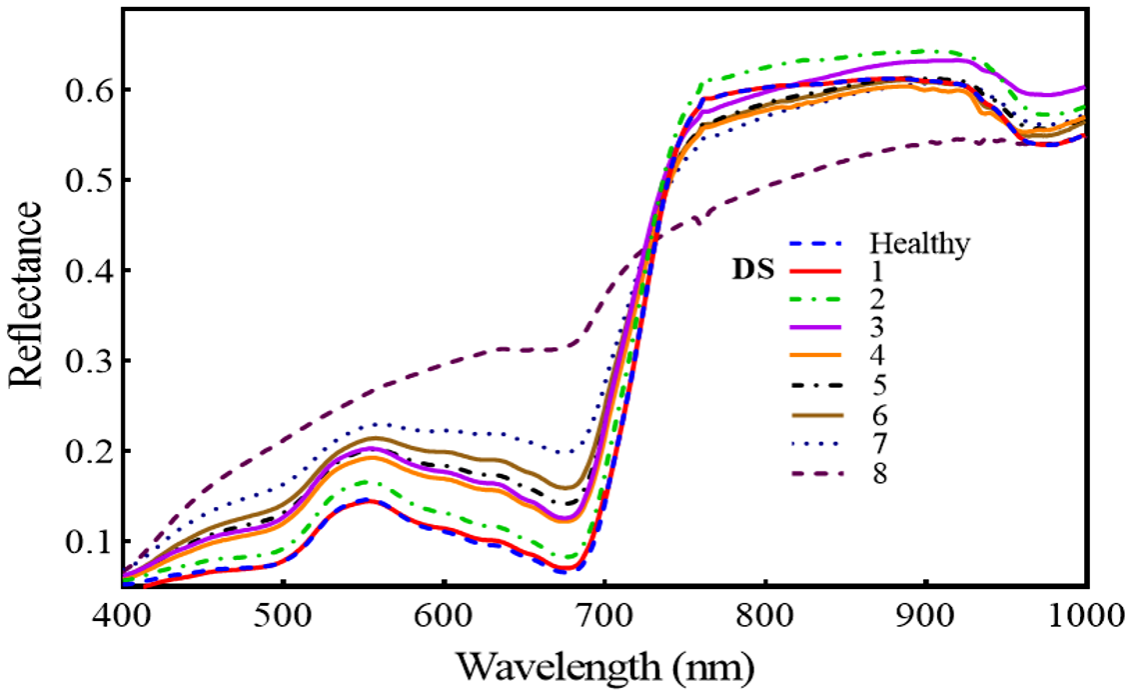
Illustrates the temporal effect of fusarium head blight (FHB) on spectral reflectance in wheat spikes at different disease severity (DS).

**Figure 7** reveals that the regions of 420–480, 540–680, and 740–860 nm are the spectral regions most highly sensitive to FHB. Moreover, the red-edge (690–730 nm) shift toward the blue region is also prominent, and both the areas and amplitude of the red-edge decreased substantially with the intensification of FHB infestation. Across all mean spectra, there was a gradual increase in the VIS region (400–700 nm), but in the NIR region, there was a continuous decrease. For the first two levels of DS, the NIR region showed an increase, but for the next DS, it decreased substantially.

### 3.2 DSF based on variable importance score (VIP)

**Figure 8:**
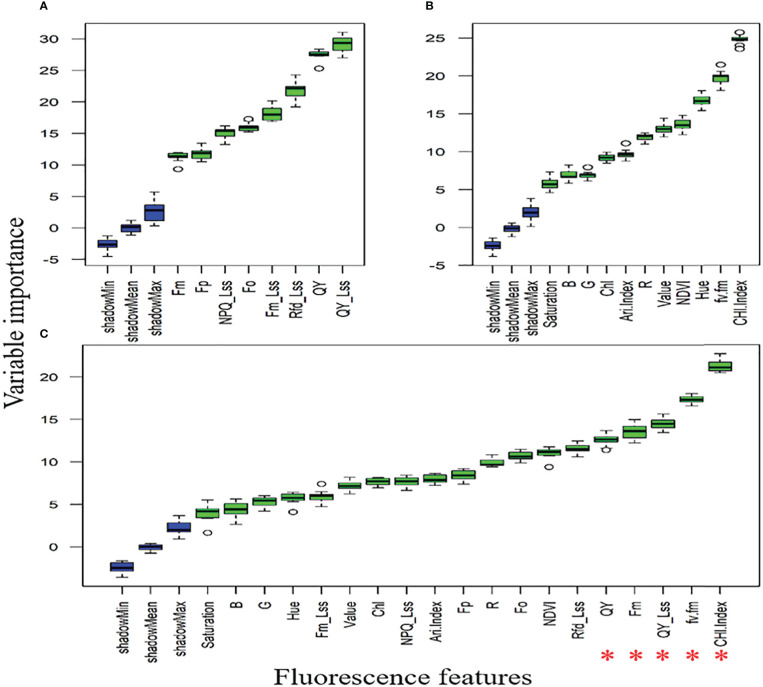
Illustration of the variable importance among studied chlorophyll fluorescence features: **(A)** comparison of the chlorophyll fluorescence imaging features **(CFI)** through variable importance score (VIP) using the pooled dataset, **(B)** comparison of fluorescence and reflectance features acquired through high-throughput phenotyping (HTP) setup through VIP using pooled dataset, **(C)** comparison of all features measured through CFI and HTP analyzed together as pooled dataset for VIP where red asterisks mark five features with high VIP.

**Figure 9:**
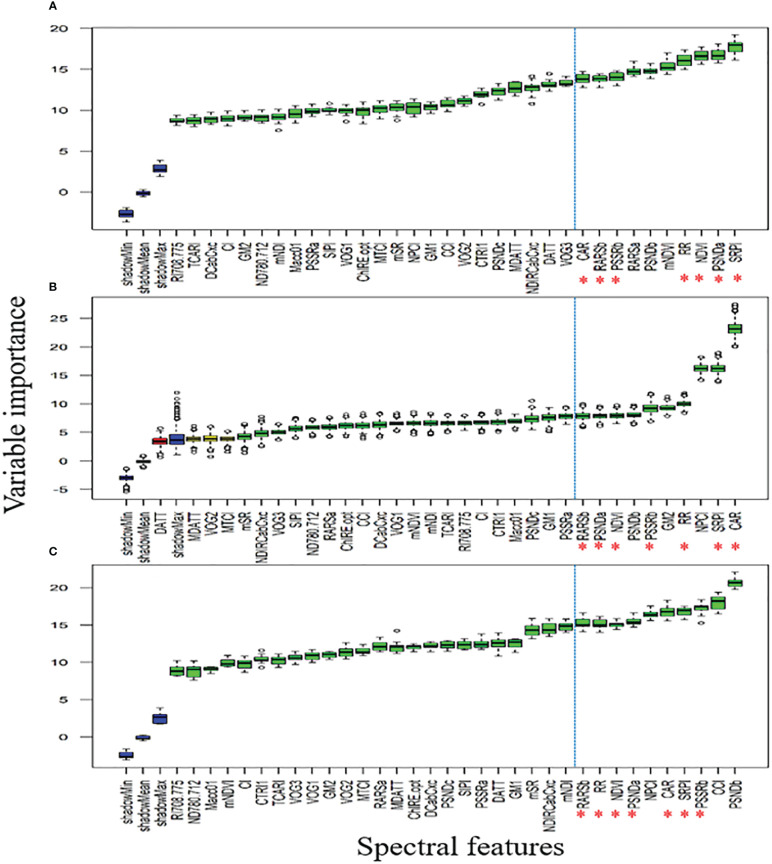
Illustration of the variable importance among studied spectral features (SF) calculated using hyperspectral reflectance: **(A)** comparison of the SF through variable importance score (VIP) using pooled dataset of first year, **(B)** comparison of the SF through VIP using pooled dataset of second year, **(C)** comparison of the SF through VIP using pooled dataset of both years.

**Figures 8A, B** showed that QY_Lss and CHL.Index are more sensitive, respectively. The top five features (CHL.Index, F_v_/F_m_, QY_Lss, F_m_, and QY) were selected with the highest VIP for subsequent analysis as DSF and marked them with red asterisks in **Figure 8C**. A pooled dataset of both periods (2019–20 and 2020–21) was analyzed and shown in **Figure 9C**. Although all of the spectral features (SF) in **Figures 9A–C** showed sensitivity to FHB, the top ten SF (partitioned by the dotted blue line) from each dataset (**Figures 9A–C**) were selected as stable and consistent DSF. Finally, only seven SF (CAR, SRPI, RR, PSSRb, NDVI, PSNDa, and RARSb) were consistent throughout **Figures 9A–C** (marked by red asterisks), which had shown consistently stable responses to FHB. The resulting DSF showing a VIF of ≤10 were retained as selected disease-specific features (SDSF) (**Table 2**).

**Table 2:** List of the disease sensitive features selected through variable importance.

	Device (Spectral meter)	Feature	Feature code
1	Chlorophyll fluorescence imaging	QY_Lss	F1
2		Fm	F2
3		QY	F3
4	High-throughput phenotyping	CHL.Index	F4*
5		Fv/Fm	F5*
6	Hyperspectral reflectance	SRPI	F6*
7		PSNDa	F7
8		NDVI	F8*
9		RR	F9*
10		PSSRb	F10
11		RARSb	F11
12		CAR	F12*

The red asterisks denote the selected disease-specific features.

### 3.3 FHB detection

**Figure 10:**
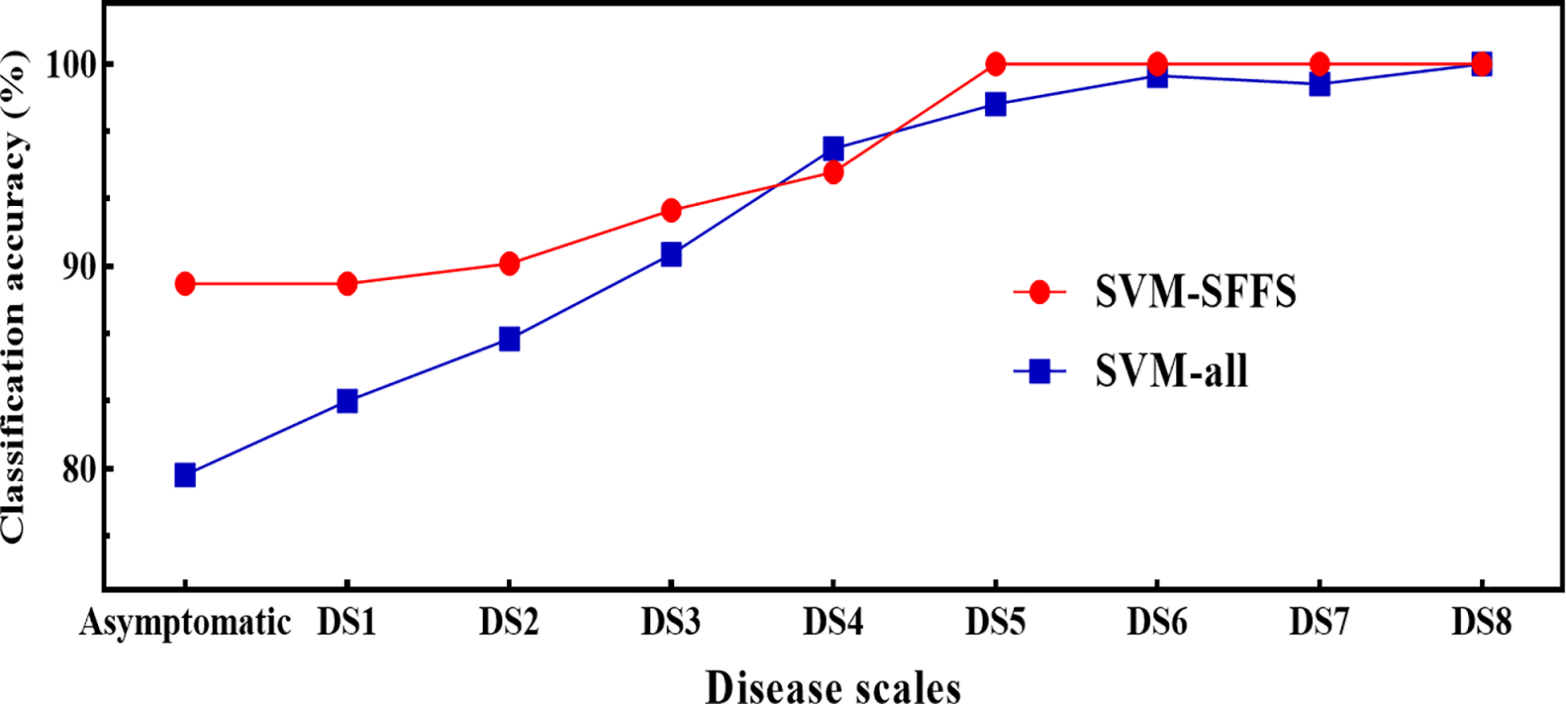
Comparison of classification accuracies of SVM between SVM-SFFS (feature selected through SFFS) and SVM-all (all selected features) using pooled dataset of two years of experiment.

Regarding the feature combination (FC), for the first five levels of DS, the combination was of two to four features but for later ones, only one to two features were sufficient to get the highest overall classification accuracy (CA) (**Table 3**). These numbers were far below than the multivariate pool of DSF. Although the FC in all three approaches were not identical, some features participated and performed significantly and consistently, i.e., F5 (F_v_/F_m_) and F8 (NDVI). **Figure 10** shows a comparison of the selected features from the SFFS and the use of all SDSF. Although the CA is satisfactory for both approaches, considerable differences prevailed. SVM-SFFS showed better CA than SVM-all, which might be due to a dimensionality factor.

**Table 3:** Evaluation of ML-SFFS for optimized feature combination (FC) to obtain the highest classification accuracy with the proliferation of disease severity from DS 1 to 8.

Disease severity	RF		K-NN		SVM	
(DS)	Feature combination (FC)	Overall classification accuracy (%)	Feature combination (FC)	Overall classification accuracy (%)	Feature combination (FC)	Overall classification accuracy (%)
Asymptomatic	F4, F9, F6	84.86	F5, F8, F6, F12	87.14	F4, F9, F8	**89.14**
DS1	F5, F8	85.24	F5, F8	88.26	F5, F8	**89.14**
DS2	F5, F6	88.01	F5, F4, F8	89.00	F5, F4, F8	**90.14**
DS3	F5, F9	92.00	F4, F8	90.09	F5, F8	**92.77**
DS4	F5, F8	**96.33**	F5, F6	94.36	F4, F6	94.66
DS5	F5	100	F5	100	F5	**100**
DS6	F5	100	F5	100	F5	**100**
DS7	F5, F4	100	F5	100	F5	**100**
DS8	F5	100	F5	100	F5, F8	**100**

the highest overall classification accuracy at each DS is highlighted in bold.

**Figure 11:**
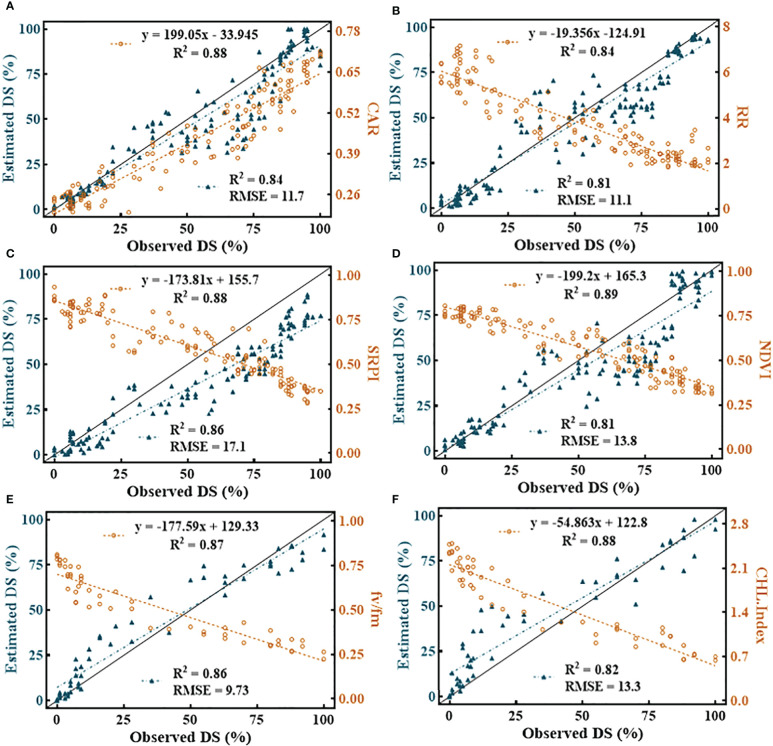
Illustration of univariate quantitative relationship among selected disease specific features (SDSF) and disease severity (DS). **(A)** carter indices (CAR), **(B)** Reciprocal Reflectance (RR), **(C)** simple ratio pigment index (SRPI), **(D)** normalized difference vegetation index (NDVI), **(E)** Photosynthetic efficiency of photosystem II (Fv/Fm) and **(F)** chlorophyll index (CHL-Index).

**Figure 12:**
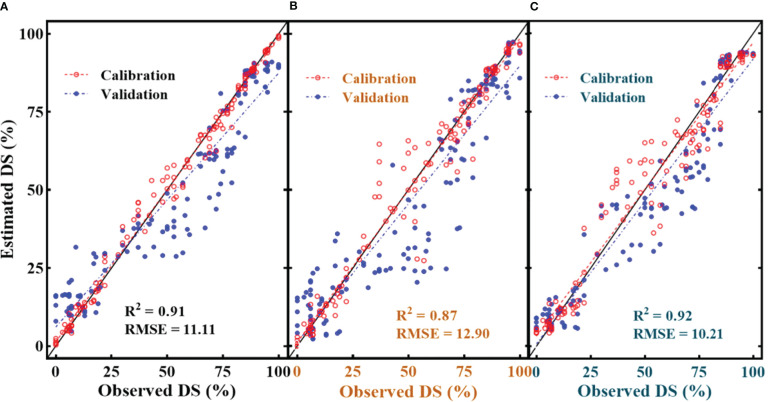
Illustration of multivariate quantitative relationship between selected disease specific features (SDSF) and disease severity (DS). **(A)** random forest regression with SDSF, **(B)** support vector machine regression with SDSF and **(C)** K-NN regression with SDSF.

Among all the SDSF, SRPI (**Figure 11C**) yielded the highest RMSE = 17.1 with *R* ^2^ = 0.86 using an estimated equation developed on the dataset with *R* ^2^ = 0.88. However, NDVI (**Figure 11D**) showed a lower RMSE = 13.8 with *R* ^2^ = 0.81, compared to SRPI, *R* ^2^ between the developed model and the cross-validated datasets exhibited a much greater difference. The minimum RMSE = 9.73 with *R* ^2^ = 0.86 (**Figure 11E**) during disease estimation was shown by F_v_/F_m_. Regarding the multivariate models, in respect of their effectiveness in the FHB estimation models, the RF model (**Figure 12A**) resulted in RMSE = 11.11 with *R* ^2^ = 0.91, the SVM model gave an RMSE = 12.90 with *R* ^2^ = 0.87, and K-NN outperformed all the others, resulting in an RMSE = 10.20 with *R* ^2^ = 0.92. Convincingly, all the SDSF explained the significant variation with DS, and model equations had the excellent predictive ability for FHB estimation.

## 4 Discussion

### 4.1 Interpretation of disease-sensitive features from different categories

FHB pathogen invasion on wheat spikes damaged the spikelets’ anatomy along with disease proliferation. This damage reduced Pn (Mustafa et al., 2022), SCC, and eventually resulted in the gradual and complete destruction of spike structure. The results confirmed this trend for Pn (**Figures 4A, B**) and SCC (**Figures 4C, D**). Given these, the reduction in the biochemical functions of wheat spikes can be attributed to pathogen development. In addition, SF, CFI and HTP features (**Figures 7**, **8**) are evident of the photosynthetic damage to the spike structure because these features are pertinent to chlorophyll-related studies (**Table 1**).

The SDSF from VIP analysis has excellent sensitivity to FHB disease, and each feature clutches specific relevance to plants’ chemistry. For instance, among the SF (SRPI, NDVI, CAR, CHL.Index), SRPI has been previously cited as being most sensitive to chlorophyll and carotenoid components (Gamon et al., 2016). Likewise, NDVI, CAR, and CHL.Index leverage support from the literature as plant pigment indices (Rouse et al., 1974). Previous studies have successfully employed the VI for disease detection in different crops (Ren et al., 2021). Accordingly, FHB detection and monitoring have investigated the VI for hyperspectral imaging and found PSSRa and PSSRb most sensitive (Bauriegel et al., 2011; Mahlein et al., 2019). This study also found these two VI sensitive to FHB disease (**Figure 9**) but failed to compete with the chosen ones on behalf of the criteria of consistent behavior and VIP. The reason might be that these studies had not adopted consistent features selection approach and claimed correlation-based sensitivity. However, this study selected SRPI, NDVI, CAR, and CHL.Index for classification using a consistent feature selection approach. Resultantly the SF have sensitivity for FHB detection and could be attributed for pigment damage in the plants. The reflectance pattern (**Figure 7**) and the development of FHB invasion severity are in accord with the findings of previous studies (Ha et al., 2016 and Huang et al., 2020) that also represent the pigment damage and red-edge shift under disease stress.

Chlorophyll fluorescence is a well-known noninvasive approach to examine the photosynthetic fingerprints of stress (biotic or abiotic) on the metabolism of plants (Gitelson et al., 1999; Ehlert and Hincha, 2008; Huang et al., 2019b). Numerous studies have reported Fv/Fm as an integral fluorescence attribute for successful plant examination under applied crop treatments (Harbinson, 2013; Lei et al., 2017). A couple of studies found F_v_/F_m_ from CFI as a strong candidate for FHB detection (Bauriegel et al., 2011; Bauriegel and Herppich, 2014), even when the symptoms were not visible on the glumes (Mahlein et al., 2019). Likewise, in this study, F_v_/F_m_ played a substantial role under VIP analysis for CFI and HTP. Since all the SDSFs were selected from the plant pigment-related studies, these could be potential candidates for studying FHB fingerprints on wheat spikes for pigment damage and detection.

### 4.2 Early detection of FHB with ML-SFFS

In contrast to ML-SFFS, a few studies have examined optimal features or feature fusion on disease detection (Zarco-Tejada et al., 2018). Ultimately, feature selection employing either approach is an effective tactic for handling the large volume of data by reducing redundant information. Hence, ML-SFFS following VIF analysis easily overcomes the collinearity challenge and also deals with the computation load (Tian et al., 2021). In former investigation, Mahlein et al. (2019) obtained no significant improvement with the sensor fusion approach after three days of disease inoculation. However, Bauriegel et al. (2011) claimed improved CA for hyperspectral and fluorescence imaging fusion. In current study at the asymptomatic scale obtained high CA and at DS1 it manifested 87% CA. Three to four features could claim the highest potential CA, which is interpreted as each feature showing variation under pathogen attack, and the overall obtained accuracy was satisfactory. The notable factor is that fluorescence features competed strongly with VI at each level of DS. In fact, over the range DS1–8, F5 shared in each classification approach (RF, K-NN, and SVM), except at DS3 in K-NN. Moreover, this ML methodology explains the interpretability and rationality of the FC, because some features might perform better at one DS than at another. For example, F5 intervened in most levels of DS compared to any other feature due to its great sensitivity to FHB (Mahlein et al., 2019) in the studied datasets. The inclusion of different features at different levels of DS also help to interpret the disease-specific responses to the specific features. For example, all spectral features showed sensitivity to FHB in the VIP algorithmic test, but few (DSF) were of more importance where further redundancy led to obtain the SDSF. However, by performing SDSF and ML-SFFS maneuvers, the most relevant features for studying the photosynthetic fingerprints of FHB for classification were selected and abundant redundancy was filtered out. Similar approaches have been adopted to determine the effective plant traits in *Xylella fastidiosa* infection (Zarco-Tejada et al., 2018).

The SDSF adjusted the combination of different features for FHB classification at different levels of DS and resulted in the best CA under ML-SFFS. Subsequently, employed the SDSF for FHB estimation by feeding into univariate and multivariate estimation modeling. Both results are examinable for proxy estimation of FHB. In comparison, multivariate estimation resulted in better accuracy than univariate models (**Figure 11**, **Figure 12**), which agrees with Zhang et al. (2014), who estimated yellow rust in wheat using wavelet features and VI.

### 4.3 Advantages of methodology

Extraction and selection of features from hyperspectral and chlorophyll fluorescence data can significantly enhance computing efficiency and highlight the essential elements for the development of classification methodologies. In contrast, feature selection algorithms have been demonstrated to be efficient for maintaining important information while reducing computation time (Huang et al., 2019a). The suggested ML-SFFS classification approach outperforms earlier disease classification models by combination of sensitive features for high CA. The significant rise in CA with DS shows that FC with two to four features could give a higher CA than all DSF with lower computational cost (**Figure 10**). Individual spectral features (Mahlein et al., 2013; Shi et al., 2018) and ML (Rumpf et al., 2010) have been used in previous studies with promising results for the detection of plant diseases (Cheng et al., 2010; Mahlein et al., 2013). However, the majority of previous studies utilized complex classification algorithms for disease detection and only a few attempts were made to enhance FC for higher classification performance (Rumpf et al., 2010; Zarco-Tejada et al., 2018). By combination of sensitive features, the weak features to disease stress at the earliest stage of infection could be successfully amplified. In this work, the application of VIF analysis and ML-SFFS algorithm enabled not only the decrease of collinearity among predictor variables, but also the reduction of computational burden.

ML-based classification ensures logic and interpretability of FC picked from SDSF by the SFFS technique. For instance, reflectance and fluorescence data characteristics may have performed well but could be uninterpretable and case-specific. However, all FC identified from SDSF were directly connected to FHB infection, physiological and morphological changes in infected spikes, allowing this methodology’s generalization and transferability to examine other diseases (Zarco-Tejada et al., 2018). Sensitive features for disease detection have been discussed in the literature recently (Poblete et al., 2020). A FC usually incorporate numerous plant attributes, which better illustrates FHB’ infection’s complicated physiological processes, and can explain this variance through spectral and fluorescence features.

### 4.4 Applications and limitations

The photosynthetic fingerprints, particularly on SCC were more sensitive than Pn under FHB pathogen invasion while several studies have demonstrated its integral role in grain filling (Tambussi et al., 2007; Jia et al., 2015). This might facilitate FHB detection on a large scale that could be challenging in the context of destructive sampling. In addition, studies have explored the VI for FHB detection (Bauriegel et al., 2011; Bauriegel and Herppich, 2014), which can be practiced with the currently available technologies. Moreover, numerous studies have resulted in efficient disease detection for other crops deploying VI (Zarco-Tejada et al., 2018; Mahlein et al., 2019; Tian et al., 2021). Despite attaining high CA at different scales, the ML-SFFS approach has revealed some key concerns over FC, the combination of different features at each scale and the inclusion of different sensor data. Consequently, this can restrict its large-scale application for disease detection because relative disease sensitivity can vary at different levels of DS. However, for disease quantification and estimation, the SDSF exhibited substantial potential for univariate and multivariate modeling. Moreover, SDSF can be employed in remotely sensed disease detection systems at different scales for deep phenotyping of wheat spikes. Hence, this disease detection methodology can be applied in different farm fields developing a manageable data acquisition setup.

## 5 Conclusion

This study explored the remotely sensed chlorophyll-related phenotypes greatly affected by FHB. Twelve highly sensitive to FHB infection features were observed from two years of experiments under non-destructive data acquisition approach. Likewise, the wheat spikes’ biochemical parameters also showed sensitivity to the spike–pathogen interaction during the study. The studied parameters were highly responsive for investigating the photosynthetic fingerprints of FHB and classification. This suggests the transferable application of practiced non-destructive disease detection methodology for the spike–pathogen interaction. The following conclusions can be drawn from this study’s results.

(1) Observation of the variable importance of the Boruta algorithm and consideration of all chlorophyll-related traits confirmed the destruction of photosynthesis under FHB pathogen invasion. Hence, the selected disease-sensitive features (SDSF) were highly responsive to FHB growth. In addition, the reflectance patterns of aggravated disease severity clearly demonstrated damage to plant pigments (gradual rise in the visible region) and spike structure (gradual fall in the near-infrared region).
(2) Overall classification accuracy was improved (Asymptomatic 87.04% to 95% at 20% disease severity) using SDSF in machine learning-sequential floating forward selection using two to four features’ combinations.
(3) Maximum univariate disease estimation was obtained through CHL.Index, and for multivariate estimation accuracy of R^2^ = 0.92 and RMSE = 10.21 through k-nearest neighbor model.

Future studies are advised to develop a more concise and decisive combination of features (disease index) in applying SDSF to other plant diseases and cultivars. The development of sensors with partial feature fusion (reflectance and fluorescence) for disease detection may also prove useful application in precision crop management both at greenhouse and field experiments.

## Data availability statement

The original contributions presented in the study are included in the article/**Supplementary Material**. Further inquiries can be directed to the corresponding authors.

## Author contributions

HJ, GL, TC, YT, WC, YZ and XY project conceptualization, supervision and administration. GM and HZ methodology, data analysis and draft writing. WL, YY, YW, MZ, PL and MB actively participated for data acquisition. TC, YZ and XY reviewed, edited and improved the manuscript. All authors contributed to the article and approved the submitted version.

## References

[B1] Abid M. Shao Y. Liu S. Wang F. Gao J. Jiang D.. (2017). Pre-drought priming sustains grain development under post-anthesis drought stress by regulating the growth hormones in winter wheat (Triticum aestivum l.). Planta 246 (3), 509–524. doi: 10.1007/s00425-017-2698-4 28526982

[B2] Araus Ortega J. L. Kefauver S. C. Zaman Allah M. Olsen M. S. Cairns J. E. (2018). Translating high throughput phenotyping into genetic gain. Trends Plant Sci. 23, 451–466. doi: 10.1016/j.tplants.2018.02.001 29555431 PMC5931794

[B3] Bauriegel E. Giebel A. Geyer M. Schmidt U. Herppich W. B. (2011). Early detection of fusarium infection in wheat using hyper-spectral imaging. Comput. Electron. Agric. 75 (2), 304–312. doi: 10.1016/j.compag.2010.12.006

[B4] Bauriegel E. Herppich W. B. (2014). Hyperspectral and chlorophyll fluorescence imaging for early detection of plant diseases, with special reference to fusarium spec. infections on wheat. Agriculture 4 (1), 32–57. doi: 10.3390/agriculture4010032

[B5] Belgiu M. Drăguţ L. (2016). Random forest in remote sensing: A review of applications and future directions. ISPRS J. Photogrammetry Remote Sens. 114, 24–31. doi: 10.1016/j.isprsjprs.2016.01.011

[B6] Bhardwaj K. Patra S. (2018). An unsupervised technique for optimal feature selection in attribute profiles for spectral-spatial classification of hyperspectral images. ISPRS J. Photogrammetry Remote Sens. 138, 139–150. doi: 10.1016/j.isprsjprs.2018.02.005

[B7] Blackburn G. A. (1998a). Quantifying chlorophylls and caroteniods at leaf and canopy scales: An evaluation of some hyperspectral approaches. Remote Sens. Environ. 66 (3), 273–285. doi: 10.1016/S0034-4257(98)00059-5

[B8] Blackburn G. A. (1998b). Spectral indices for estimating photosynthetic pigment concentrations: a test using senescent tree leaves. Int. J. Remote Sens. 19 (4), 657–675. doi: 10.1080/014311698215919

[B9] Carter G. A. (1994). Ratios of leaf reflectances in narrow wavebands as indicators of plant stress. Remote Sens. 15 (3), 697–703. doi: 10.1080/01431169408954109

[B10] Chang T.-G. Song Q. F. Zhao H. L. Chang S. Xin C. Qu M.. (2020). An *in situ* approach to characterizing photosynthetic gas exchange of rice panicle. Plant Methods 16 (1), 1–14. doi: 10.1186/s13007-020-00633-1 32647532 PMC7336644

[B11] Chang C.-C. Lin C.-J. (2001). Training v-support vector classifiers: theory and algorithms. Neural Comput. 13 (9), 2119–2147. doi: 10.1162/089976601750399335 11516360

[B12] Chappelle E. W. Kim M. S. McMurtrey Iii J. E. (1992). Ratio analysis of reflectance spectra (RARS): an algorithm for the remote estimation of the concentrations of chlorophyll a, chlorophyll b, and carotenoids in soybean leaves. Remote Sens. Environ. 39 (3), 239–247. doi: 10.1016/0034-4257(92)90089-3

[B13] Cheng T. Rivard B. Sánchez-Azofeifa G. A. Feng J. Calvo-Polanco M. (2010). Continuous wavelet analysis for the detection of green attack damage due to mountain pine beetle infestation. Remote Sens. Environ. 114, 899–910. doi: 10.1016/j.rse.2009.12.005

[B14] Easlon H. M. Bloom A. J. (2014). Easy leaf area: Automated digital image analysis for rapid and accurate measurement of leaf area. Appl. Plant Sci. 2 (7), 1400033. doi: 10.3732/apps.1400033 PMC4103476 25202639

[B15] Ehlert B. Hincha D. K. (2008). Chlorophyll fluorescence imaging accurately quantifies freezing damage and cold acclimation responses in arabidopsis leaves. Plant Methods 4 (1), 1–7. doi: 10.1186/1746-4811-4-12 18505561 PMC2430023

[B16] Fallahpour S. Lakvan E. N. Zadeh M. H. (2017). Using an ensemble classifier based on sequential floating forward selection for financial distress prediction problem. J. Retailing Consumer Serv. 34, 159–167. doi: 10.1016/j.jretconser.2016.10.002

[B17] Gamon J. A. Huemmrich K. F. Wong C. Y. S. Ensminger I. Garrity S. Hollinger D. Y.. (2016). A remotely sensed pigment index reveals photosynthetic phenology in evergreen conifers. Proc. Natl. Acad. Sci. 113 (46), 13087–13092. doi: 10.1073/pnas.1606162113 27803333 PMC5135292

[B18] Gitelson A. A. Buschmann C. Lichtenthaler H. K. (1999). The chlorophyll fluorescence ratio F735/F700 as an accurate measure of the chlorophyll content in plants. Remote Sens. Environ. 69 (3), 296–302. doi: 10.1016/S0034-4257(99)00023-1

[B19] Gong P. Pu R. Heald R. C. (2002). Analysis of *in situ* hyperspectral data for nutrient estimation of giant sequoia. Int. J. Remote Sens. 23 (9), 1827–1850. doi: 10.1080/01431160110075622

[B20] Granum E. Pérez-Bueno M. L. Calderón C. E. Ramos C. de Vicente A. Cazorla F. M.. (2015). Metabolic responses of avocado plants to stress induced by rosellinia necatrix analysed by fluorescence and thermal imaging. Eur. J. Plant Pathol. 142 (3), 625–632. doi: 10.1007/s10658-015-0640-9

[B21] Ha X. Koopmann B. Von Tiedemann A. (2016). Wheat blast and fusarium head blight display contrasting interaction patterns on ears of wheat genotypes differing in resistance. Phytopathology 106, 270–281. doi: 10.1094/PHYTO-09-15-0202-R 26574785

[B22] Harbinson J. (2013). Improving the accuracy of chlorophyll fluorescence measurements. Plant Cell Environ. 36 (10), 1751–1754. doi: 10.1111/pce.12111 23560881

[B23] Huang L. Wu Z. Huang W. Ma H. Zhao J. (2019a). Identification of fusarium head blight in winter wheat ears based on fisher’s linear discriminant analysis and a support vector machine. Appl. Sci. 9 (18), 3894. doi: 10.3390/app9183894

[B24] Huang Y. Li Z. Risinger A. L. Enslow B. T. Zeman C. J. Gong J.. (2019b). Fluorescence spectral shape analysis for nucleotide identification. Proc. Natl. Acad. Sci. 116 (31), 15386–15391. doi: 10.1073/pnas.1820713116 31308243 PMC6681718

[B25] Huang L. Li T. Ding C. Zhao J. Zhang D. Yang G. (2020). Diagnosis of the severity of fusarium head blight of wheat ears on the basis of image and spectral feature fusion. Sensors 20, 2887. doi: 10.3390/s20102887 32443656 PMC7287655

[B26] Jia S. Lv J. Jiang S. Liang T. Liu C. Jing Z. (2015). Response of wheat ear photosynthesis and photosynthate carbon distribution to water deficit. Photosynthetica 53, 95–109.

[B27] Jin X. Jie L. Wang S. Qi H. J. Li S. W. (2018). Classifying wheat hyperspectral pixels of healthy heads and fusarium head blight disease using a deep neural network in the wild field. Remote Sens. 10 (3), 395. doi: 10.3390/rs10030395

[B28] Kheiri A. Jorf S. A. M. Malihipour A. (2019). Infection process and wheat response to fusarium head blight caused by fusarium graminearum. Eur. J. Plant Pathol. 153 (2), 489–502. doi: 10.1007/s10658-018-1576-7

[B29] Kuenzer C. Knauer K. (2013). Remote sensing of rice crop areas. Int. J. Remote Sens. 34 (6), 2101–2139. doi: 10.1080/01431161.2012.738946

[B30] Kursa M. B. Rudnicki W. R. (2010). Feature selection with the boruta package: Journal. J. Stat. Softwar. 36, 1–13. doi: 10.18637/jss.v036.i11

[B31] Lei R. Jiang H. Hu F. Yan J. Zhu S. (2017). Chlorophyll fluorescence lifetime imaging provides new insight into the chlorosis induced by plant virus infection. Plant Cell Rep. 36 (2), 327–341. doi: 10.1007/s00299-016-2083-y 27904946

[B32] Lichtenthaler H. K. (1987). Chlorophylls and carotenoids: pigments of photosynthetic biomembranes. Methods enzymology 148, 350–382. doi: 10.1016/0076-6879(87)48036-1

[B33] Long Y. Rivard B. Rogge D. Tian M. (2019). Hyperspectral band selection using the n-dimensional spectral solid angle method for the improved discrimination of spectrally similar targets. Int. J. Appl. Earth Observation Geoinformation 79, 35–47. doi: 10.1016/j.jag.2019.03.002

[B34] Ma H. Huang W. Jing Y. Pignatti S. Laneve G. Dong Y.. (2020). Identification of fusarium head blight in winter wheat ears using continuous wavelet analysis. Sensors 20 (1), 20. 10.3390/s20010020 PMC6982701 31861503

[B35] Mahlein A. K. Rumpf T. Welke P. Dehne H. W. Plümer L. Steiner U.. (2013). Development of spectral indices for detecting and identifying plant diseases. Remote Sens. Environ. 128, 21–30. doi: 10.1016/j.rse.2012.09.019

[B36] Mahlein A.-K. Kuska M. T. Thomas S. Wahabzada M. Behmann J. Rascher U.. (2019). Comparison and combination of thermal, fluorescence, and hyperspectral imaging for monitoring fusarium head blight of wheat on spikelet scale. Sensors 19 (10), 2281. doi: 10.3390/s19102281 31108868 PMC6567885

[B37] McBeath J. H. McBeath J. (2010). “Plant diseases, pests and food security,” in Environmental Change and Food Security in China (Springer) 117–156.

[B38] Mehta P. Jajoo A. Mathur S. Bharti S. (2010). Chlorophyll a fluorescence study revealing effects of high salt stress on photosystem II in wheat leaves. Plant Physiol. Biochem. 48 (1), 16–20. doi: 10.1016/j.plaphy.2009.10.006 19932973

[B39] Meng L. Mestdagh H. Ameye M. Audenaert K. Höfte M. Van Labeke M.C.. (2020). Phenotypic variation of botrytis cinerea isolates is influenced by spectral light quality. Front. Plant Sci. 11, 1233. doi: 10.3389/fpls.2020.01233 32903526 PMC7438557

[B40] Mustafa G. Zheng H. Khan I. H. Tian L. Jia H. Li G.. (2022). Hyperspectral reflectance proxies to diagnose in-field fusarium head blight in wheat with machine learning. Remote Sens. 14, 2784. doi: 10.1109/TGRS.2022.3178125

[B41] Oumar Z. Mutanga O. Ismail R. (2013). Predicting thaumastocoris peregrinus damage using narrow band normalized indices and hyperspectral indices using field spectra resampled to the Hyperion sensor. Int. J. Appl. Earth observation Geoinformation 21, 113–121. doi: 10.1016/j.jag.2012.08.006

[B42] Pineda M. Gáspár L. Morales F. Szigeti Z. Baron M. (2008). Multicolor fluorescence imaging of leaves–a useful tool for visualizing systemic viral infections in plants. Photochem. Photobiol. 84 (5), 1048–1060. doi: 10.1111/j.1751-1097.2008.00357.x 18435702

[B43] Poblete T. Camino C. Beck P. S. A. Hornero A. Kattenborn T. Saponari M.. (2020). Detection of xylella fastidiosa infection symptoms with airborne multispectral and thermal imagery: Assessing bandset reduction performance from hyperspectral analysis. ISPRS J. Photogrammetry Remote Sens. 162, 27–40. doi: 10.1016/j.isprsjprs.2020.02.010

[B44] Pudil P. Novovičová J. Kittler J. (1994). Floating search methods in feature selection. Pattern recognition Lett. 15 (11), 1119–1125. doi: 10.1016/0167-8655(94)90127-9

[B45] Ren Y. Huang W. Ye H. Zhou X. Ma H. Dong Y.. (2021). Quantitative identification of yellow rust in winter wheat with a new spectral index: Development and validation using simulated and experimental data. Int. J. Appl. Earth Observation Geoinformation 102, 102384. doi: 10.1016/j.jag.2021.102384

[B46] Rouse J. W. Haas R. H. Schell J. A. Deering D. W. Harlan J. C. (1974). “Monitoring the vernal advancement and retrogradation (green wave effect) of natural vegetation,” in NASA/GSFC type III final report (Greenbelt, Md) 371.

[B47] Rumpf T. Mahlein A. K. Steiner U. Oerke E. C. Dehne H. W. Plümer L. (2010). Early detection and classification of plant diseases with support vector machines based on hyperspectral reflectance. Computers and Electronics in Agriculture 74, 91–99.

[B48] Shi Y. Huang W. Ye H. Ruan C. Xing N. Geng Y.. (2018). Partial least square discriminant analysis based on normalized two-stage vegetation indices for mapping damage from rice diseases using PlanetScope datasets. Sensors 18, 1901. doi: 10.3390/s18061901 29891814 PMC6021985

[B49] Stack R. W. McMullen M. P. (1998). A visual scale to estimate severity of fusarium head blight in wheat.

[B50] Tambussi E. A. Bort J. Guiamet J. J. Nogués S. Araus J. L. (2007). The photosynthetic role of ears in C3 cereals: metabolism, water use efficiency and contribution to grain yield. Crit. Rev. Plant Sci. 26 (1), 1–16. doi: 10.1080/07352680601147901

[B51] Tan J. De Zutter N. De Saeger S. De Boevre M. Tran T. M. van der Lee T.. (2021). Presence of the weakly pathogenic fusarium poae in the fusarium head blight disease complex hampers biocontrol and chemical control of the virulent fusarium graminearum pathogen. Front. Plant Sci. 12, 216. doi: 10.3389/fpls.2021.641890 PMC7928387 33679858

[B52] Thenkabail P. S. Enclona E. A. Ashton M. S. van der Meer B. (2004). Accuracy assessments of hyperspectral waveband performance for vegetation analysis applications. Remote Sens. Environ. 91 (3-4), 354–376. doi: 10.1016/j.rse.2004.03.013

[B53] Tian L. Xue B. Wang Z. Li D. Yao X. Cao Q.. (2021). Spectroscopic detection of rice leaf blast infection from asymptomatic to mild stages with integrated machine learning and feature selection. Remote Sens. Environ. 257, 112350. doi: 10.1016/j.rse.2021.112350

[B54] Wei R. Ye C. Sui T. Ge Y. Li Y. Li J.. (2022). Combining spatial response features and machine learning classifiers for landslide susceptibility mapping. Int. J. Appl. Earth Observation Geoinformation 107, 102681. doi: 10.1016/j.jag.2022.102681

[B55] Weinberger K. Q. Blitzer J. Saul L. K. (2006). Distance metric learning for large margin nearest neighbor classification 1473–1480.

[B56] Zarco-Tejada P. J. Camino C. Beck P. S. A. Calderon R. Hornero A. Hernández-Clemente R.. (2018). Previsual symptoms of xylella fastidiosa infection revealed in spectral plant-trait alterations. Nat. Plants 4 (7), 432–439. doi: 10.1038/s41477-018-0189-7 29942047

[B57] Zhang D.-Y. Chen G. Yin X. Hu R. J. Gu C.-Y. Pan Z.-G.. (2020). Integrating spectral and image data to detect fusarium head blight of wheat. Comput. Electron. Agric. 175, 105588. doi: 10.1016/j.compag.2020.105588

[B58] Zhang J. Pu R. Loraamm R. W. Yang G. Wang J. (2014). Comparison between wavelet spectral features and conventional spectral features in detecting yellow rust for winter wheat. Comput. Electron. Agric. 100, 79–87. doi: 10.1016/j.compag.2013.11.001

[B59] Zhao X. Hou Z. Wu X. Li W. Ma P. Tao R. (2021). Hyperspectral target detection based on transform domain adaptive constrained energy minimization. Int. J. Appl. Earth Observation Geoinformation 103, 102461. doi: 10.1016/j.jag.2021.102461

