# Supplementary material for: Fusarium head blight monitoring in wheat ears using machine learning and multimodal data from asymptomatic to symptomatic periods
Source: Front Plant Sci. 2023 Jan 16;13:1102341. doi: 10.3389/fpls.2022.1102341 (PMC9885105; doi:10.3389/fpls.2022.1102341)
Supplement: Supplementary file 1 [file Table_1.docx]

Fusarium head blight monitoring in wheat ears using machine learning and multimodal data during asymptomatic to symptomatic periods

|  | **Year** | **Cultivars** | **Number of pots** | **Data type** |
| --- | --- | --- | --- | --- |
| Exp. 1 | 2019–2020 | Aikang-58 and Sumai-3 | 24 | Hyperspectral reflectance  Chlorophyll fluorescence imaging |
| Exp. 2 | 2020–2021 | Aikang-58 and Sumai-3 | 24 | Hyperspectral reflectance  Chlorophyll fluorescence imaging |
| Exp. 3 | 2020–2021 | Aikang-58, Bainong-418, Zhongyou-9507, Jimai-31, Wenmai-6, Chianmai-42, Huangpei-R4, and Sumai-3 | 56 | High throughput phenotyping |

**Table S1** Detail of the plant material

**Table S2** Spectral chlorophyll related indices selected for fusarium head blight detection and estimation in current study

|  | **Chlorophyll indices** | **Abbreviations** | **Formulas** | **References** |
| --- | --- | --- | --- | --- |
| **1** | Reciprocal Reflectance | RR | $1/R_{700}$ | Gitelson et al. (1999) |
| **2** | Modified Red-edge Ratio | mSR | (R_750_-R_445_)/(R_705_-R_445_) | Sims and Gamon (2002) |
| **3** | Pigment Specific Simple Ratio | PSSRa | R_800_/R_675_ | Blackburn (1998b) |
| **4** |  | PSSRb | R_800_/R_650_ | Blackburn (1999) |
| **5** | Ratio Analysis of Reflectance Spectra | RARSa | R_675_/R_700_ | Chappelle et al. (1992) |
| **6** |  | RARSb | R_675_/(R_675_×R_700_) |  |
| **7** | Normalized Difference Vegetation Index | NDVI | (R800-R670)/(R800+R670) | Rouse et al. (1974) |
| **8** | Red-edge NDVI | mNDVI | (R_750_-R_705_)/  (R_750_+R_705_) | Gitelson and Merzlyak (1994) |
| **9** | Modified Red-edge Normalized Difference Vegetation Index | mNDI | (R_750_-R_705_)/(R_750_-R_705_-2R_445_) | Sims and Gamon (2002) |
| **10** | Pigment Specific Normalized Difference | PSNDa | (R_800_-R_675_)/  (R_800_+R_675_) | Blackburn (1998a) |
| **11** |  | PSNDb | (R_800_-R_650_)/  (R_800_+R_650_) |  |
| **12** |  | PSNDc | (R_800_-R_470_)/  (R_800_+R_470_) |  |
| **13** | Macc01 | Macc01 | (R_780_-R_710_)/(R_780_-R_680_) | Maccioni et al. (2001) |
| **14** | The MERIS terrestrial chlorophyll ind | MTCI | (R_754_-R_709_)/(R_709_-R_681_) | Dash and Curran (2004) |
| **15** | DATT | DATT | (R_850_-R_710_)/(R_850_-R_680_) | Datt (1998) |
| **16** | Modified DATT | MDATT | (R_721_-R_744_)/(R_721_-R_714_) | Datt (1999) |
| **17** | Vogelmann indices | VOG1 | R_740_/R_720_ | Vogelmann et al. (1993) |
| **18** |  | VOG2 | (R_734_-_747_)/(R_715_-_726_) |  |
| **19** |  | VOG3 | (R_734_-R_747_)/(R_715_+R_720_) |  |
| **20** | Gitelson & Merzlyak indices | GM1 | R_750_/R_550_ | Gitelson and Merzlyak (1996) |
| **21** |  | GM2 | R_750_/R_700_ |  |
| **22** | Transformed Chlorophyll Absorption in Reflectance Index | TCARI | 3×[(R700-R670)-0.2×(R700-R550)× (R700/R670)] | Haboudane et al. (2002) |
| **23** | Chlorophyll Index Red Edge | CI | R_750_/R_710_ |  |
| **24** | Simple Ratio Pigment Index | SRPI | R_430_/R_680_ | Penuelas et al. (1995) |
| **25** | Normalized Pigments Index | NPCI | (R_680_-R_430_)/(R_680_+R_430_) |  |
| **26** | Carter indices | CTRI1 | R_695_/R_420_ | Carter (1994) |
| **27** |  | CAR | R_695_/R_760_ |  |
| **28** | Reflectance band ratio indices | DCabCxc | R672/(R550×3R708) | Datt (1998) |
| **29** |  | NDIRCabCxc | R_860_/(R_550_×R_708_) |  |
| **30** | Structure-Intensive Pigment Index | SIPI | (R_800_-R_445_)/(R_800_+R_680_) | Penuelas et al. (1995) |
| **31** | ChlRE opt | ChlRE opt | (1/R_680-730_-1/R_780-800_)×R_755-780_ | Féret et al. (2011) |
| **32** | RI_708,775_ | RI708,775 | R_708_/R_775_ |  |
| **33** | ND_780,712_ | ND780,712 | (R_780_-R_712_)/(R_780_+R_712_) |  |
| **34** | Chlorophyll/carotenoid Index | CCI | (R_531_-R_645_)/(R_531_+R_645_) | Gamon et al. (2016) |

**References**

Blackburn, G.A., 1998a. Quantifying chlorophylls and caroteniods at leaf and canopy scales: An evaluation of some hyperspectral approaches. Remote Sensing of Environment 66, 273-285.

Blackburn, G.A., 1998b. Spectral indices for estimating photosynthetic pigment concentrations: a test using senescent tree leaves. International Journal of Remote Sensing 19, 657-675.

Blackburn, G.A., 1999. Relationships between spectral reflectance and pigment concentrations in stacks of deciduous broadleaves. Remote Sensing of Environment 70, 224-237.

Carter, G.A., 1994. Ratios of leaf reflectances in narrow wavebands as indicators of plant stress. Remote Sensing 15, 697-703.

Chappelle, E.W., Kim, M.S., McMurtrey Iii, J.E., 1992. Ratio analysis of reflectance spectra (RARS): an algorithm for the remote estimation of the concentrations of chlorophyll a, chlorophyll b, and carotenoids in soybean leaves. Remote Sensing of Environment 39, 239-247.

Dash, J., Curran, P.J., 2004. The MERIS terrestrial chlorophyll index.

Datt, B., 1998. Remote sensing of chlorophyll a, chlorophyll b, chlorophyll a+ b, and total carotenoid content in eucalyptus leaves. Remote Sensing of Environment 66, 111-121.

Datt, B., 1999. A new reflectance index for remote sensing of chlorophyll content in higher plants: tests using Eucalyptus leaves. Journal of Plant Physiology 154, 30-36.

Féret, J.-B., François, C., Gitelson, A., Asner, G.P., Barry, K.M., Panigada, C., Richardson, A.D., Jacquemoud, S., 2011. Optimizing spectral indices and chemometric analysis of leaf chemical properties using radiative transfer modeling. Remote Sensing of Environment 115, 2742-2750.

Gamon, J.A., Huemmrich, K.F., Wong, C.Y.S., Ensminger, I., Garrity, S., Hollinger, D.Y., Noormets, A., Peñuelas, J., 2016. A remotely sensed pigment index reveals photosynthetic phenology in evergreen conifers. Proceedings of the National Academy of Sciences 113, 13087-13092.

Gitelson, A., Merzlyak, M.N., 1994. Quantitative estimation of chlorophyll-a using reflectance spectra: Experiments with autumn chestnut and maple leaves. Journal of Photochemistry and Photobiology B: Biology 22, 247-252.

Gitelson, A.A., Buschmann, C., Lichtenthaler, H.K., 1999. The chlorophyll fluorescence ratio F735/F700 as an accurate measure of the chlorophyll content in plants. Remote Sensing of Environment 69, 296-302.

Gitelson, A.A., Merzlyak, M.N., 1996. Signature analysis of leaf reflectance spectra: algorithm development for remote sensing of chlorophyll. Journal of plant physiology 148, 494-500.

Haboudane, D., Miller, J.R., Tremblay, N., Zarco-Tejada, P.J., Dextraze, L., 2002. Integrated narrow-band vegetation indices for prediction of crop chlorophyll content for application to precision agriculture. Remote Sensing of Environment 81, 416-426.

Maccioni, A., Agati, G., Mazzinghi, P., 2001. New vegetation indices for remote measurement of chlorophylls based on leaf directional reflectance spectra. Journal of Photochemistry and Photobiology B: Biology 61, 52-61.

Penuelas, J., Baret, F., Filella, I., 1995. Semi-empirical indices to assess carotenoids/chlorophyll a ratio from leaf spectral reflectance. Photosynthetica 31, 221-230.

Rouse, J.W., Haas, R.H., Schell, J.A., Deering, D.W., Harlan, J.C., 1974. Monitoring the vernal advancement and retrogradation (green wave effect) of natural vegetation. NASA/GSFC Type III Final Report, Greenbelt, Md 371.

Sims, D.A., Gamon, J.A., 2002. Relationships between leaf pigment content and spectral reflectance across a wide range of species, leaf structures and developmental stages. Remote Sensing of Environment 81, 337-354.

Vogelmann, J.E., Rock, B.N., Moss, D.M., 1993. Red edge spectral measurements from sugar maple leaves. Remote Sensing 14, 1563-1575.
